# The Set of Priors Related Concepts Instrumental in Understanding Conscious Perception Begs Clarification

**DOI:** 10.3389/fpsyg.2020.01293

**Published:** 2020-06-23

**Authors:** Talis Bachmann

**Affiliations:** Faculty of Social Sciences, University of Tartu, Tartu, Estonia

**Keywords:** consciousness, predictive coding, prior, expectation, perception, illusory perception, hallucination

More than 20 years ago, Block (1995) famously called “consciousness” a “mongrel concept.” Indeed, the same term had been used for many different things, leading to much misunderstanding and controversy in consciousness research. As it appears, the currently massively used set of priors related concepts such as “prior,” “prediction,” “expectations,” “attention,” etc. likewise hide premises for multiple parallel interpretations of the same data or misattributions to various constituent underlying mechanisms. This makes debates and discussions less fruitful than they could be when the terminology would be more carefully used.

Let me first provide briefly the published research background for the problem. Prior information processed before an actual stimulus-event has been shown to influence whether and how this stimulus event will be explicitly perceived (Friston, 2005; Hohwy, 2013; Auksztulewicz et al., 2018; Chao et al., 2018; de Lange et al., 2018; Keller and Mrsic-Flogel, 2018; Andersen et al., 2019; Berggren and Eimer, 2019; Crawford et al., 2019; Gandolfo and Downing, 2019; Hutchinson and Barrett, 2019; Lumaca et al., 2019; Meijs et al., 2019; Pennartz et al., 2019; Stefanics et al., 2019; Whyte, 2019; Wokke and Ro, 2019; Wolfe, 2019). The effects span across facilitation, inhibition, illusory distortion, and hallucination (Buschman and Miller, 2007; Aru and Bachmann, 2017a; Powers et al., 2017; Aru et al., 2018; Brascamp et al., 2018; Corlett et al., 2018; Flounders et al., 2019; Harrison and Rideaux, 2019; Hullfish et al., 2019; Tappin and Gadsby, 2019; Tulver et al., 2019; Valton et al., 2019; Varrier et al., 2019). Influence of priors may originate from long term memory or from actual perceptual processing of the information preceding the imperative stimulus-event to be identified, recognized, or psychophysically evaluated. However, the known phenomenology and behavioral nomenclature of the effects of priors has been explained by a multitude of concepts which are not necessarily mutually exclusive and bear complex interrelations, therefore creating confusion and contradictory standpoints in the theoretical discourse of consciousness science.

To illustrate the point, in Table 1 some pairs of mutually related concepts are presented, indicating mutual overlap where it appears to be present (in this paper I restrict the discourse to research where typical trial-by-trial stimuli presentation is used and responses of participants are recorded in order to measure objective veridicality and/or subjective evaluation of the stimuli). The effects of mechanisms attributed to information processing under the umbrella of one such concept are possibly caused or partly influenced by the mechanisms covered by some other such concept. It should be clear that in order to have clear-cut crispiness and validity of interpretations of the experimental results, experimental designs must allow avoiding hidden confounds or, at least, these possible confounds must be listed in the limitations part of studies (the standard requirement to stick to one concept and avoid unexplained parallel use of different concepts for the same effect goes without saying). The large range of overlap between the underlying perceptual-cognitive mechanisms signified by the different corresponding concepts in Table 1 shows how easy it is to create scientific misunderstanding, if not invalid interpretations.

**Table 1:** A set of interrelated concepts referring to the hypothetical effects of prior processed information on processing of the subsequently presented information for its perception.

	**Prior**	**Prediction**	**Prime**	**Expectation**	**Attention (top-down)**	**Search (target-)**	**Pre-cue**	**Set (mental set)**	**Anticipation**	**Conditioning (associative)**
	**1**	**2**	**3**	**4**	**5**	**6**	**7**	**8**	**9**	**10**
1. Prior	0	x	x	x	x	x	x/e	x	x	x
2. Prediction	x	0	e	e	e	e	e	x	x/e	e
3. Prime	x	e	0	e	e	e	x/e	x	e	X
4. Expectation	x	e	e	0	x/e	e	e	x/e	e	e
5. Attention (top-down)	x	e	e	e	0	x	x	x/e	e	e
6. Search target	x	e	e	e	x	0	x/e	x	e	e
7. Pre-cue	x/e	e	x/e	e	x	x/e	0	x	e	e
8. Set (mental set)	x	x	x	x/e	x/e	x	x	0	x	x
9. Anticipation	x	x/e	e	e	e	e	e	x	0	x/e
10. Conditioning (associative) stimulus	x	e	x	e	e	e	e	x	x/e	0

*When pairs of different concepts may be used as synonyms, the respective intersection in the table rows/columns is marked by x. When pairs of different concepts allow to assume the possibility of an effect from one member of the pair on the other, the respective intersection is marked by e (some possible related concepts such as forecast, foresight, readiness, preparation, demand, and adaptation have been left out of the table)*.

For example, we encounter a synonymous use of concepts together with avoidance of use of a possible alternative causal mechanism leading to the experimental results (Blom et al., 2020). The authors reported an effect of the expected position of a subsequent stimulus on the EEG markers of representing the expected stimulus even if its sensory signals were not presented in part of the trials. They used alternatedly concepts like “prior,” “prediction,” “expectation,” “anticipation” (our Table intersections 1 × 2, 1 × 4, 1 × 9), but avoided “attention” (4 × e5), although the spatial-attention mechanism may have been either causally involved or representing a hidden confound. Rungratsameetaweemana et al. (2018) did not use “prior,” but used “expectation,” which was for some reason contrasted with “attention” (suggesting replacement of the Table entry 4 × e5 by 0). Another example comes from Coll et al. (2020). They used frequency tagging methods to record EEG markers of processing alternative visual stimuli. Participants (with varying levels of autistic traits) were individually conditioned to expect one of the stimuli more than the other in the scrambling/unscrambling oscillating sequence. Relative weight of the expectation related top-down signals in the EEG markers decreased with increase in autistic traits. Concepts of “prediction” and “prior experience” were used (1 × 2), but “anticipation” or “conditioning” were not used (1 × 9, 2x/e9, 1 × 10, 2e10).

Obviously, the pattern of possible confounds and hidden sources of misinterpretation becomes even more complex and intricate when we distinguish between pre-conscious (unconscious) and conscious levels of sources of the effects related to most of the table entries. Also, it is realistic to bear in mind that no single study, even if based on many experiments, would be capable of providing finalized valid explanations for priors related phenomena and behavioral/subjective regularities of effects (likewise, it has been shown that multidimensional, brainwide activity is what drives sensory-perceptual processes—Stringer et al., 2019). Therefore, collective research efforts are recommended, capable of disentangling the different priors related causal factors, with division of tasks well thought through and agreed upon between the participating labs (consortia). At the level of single lab efforts the main recommendation would be to use clever converging operations (orthogonal factorial design) planned so as to take apart contributions of different levels and types of priors. Some studies can be mentioned as examples of such approaches (e.g., Oxner et al., 2019; Zuanazzi and Noppeney, 2019; see also Shalev et al., 2019). Some other authors have drawn attention to additional important distinctions if we want to have reliable predictive coding related theories: a model must be able to (i) explain how the often incompatible tasks of reflecting veridicality of the actual environment and conveying meaningful context dependent information are solved within this same model (Larkum, 2013; Press et al., 2020); (ii) account for both, long term priors related effects and the effects of priors involved in ongoing processing (Aru and Bachmann, 2017b; White, 2018); (iii) in vision as well as auditory perception, one of the priority tasks for developing valid models must be to distinguish attention effects from other predictive prior effects (e.g., Aru et al., 2018; Alilović et al., 2019; Kompus et al., in press). There have been also promising attempts to differentiate between types of priors such as structural and contextual expectations (Seriès and Seitz, 2013). Yet, the task of disentangling the varieties of priors is complicated as there seems to be no general factor of the effects of priors on perception (Tulver et al., 2019). On the other hand, this may be caused just by the fact that *priors* is a mongrel concept.

Understandably, there could be an explosion of differentiated concepts if we admit that even some of the subordinate level concepts themselves are “mongrels” (for “attention” this is reflected in some recommendations to abandon this concept for good—Anderson, 2011; Hommel et al., 2019. Alternatively, this concept could well be taken apart—Luo and Maunsell, 2019). Yet the main call inherent in this paper remains. Therefore, I guess many would like to see that in the papers dealing with predictive coding the “mongrel” concepts will be taken apart and the (possible) mutual effects and levels of action of the constituent mechanisms become clearly separated and specified. But even before this, a conceptual taxonomy is in the waiting. Who could accomplish the task? We have a lot of prior knowledge about the likely candidates, or have we?

## Author Contributions

The author confirms being the sole contributor of this work and has approved it for publication.

## Conflict of Interest

The author declares that the research was conducted in the absence of any commercial or financial relationships that could be construed as a potential conflict of interest.
